# Spatial variation in adult sex ratio across multiple scales in the invasive golden apple snail, *Pomacea canaliculata*

**DOI:** 10.1002/ece3.2043

**Published:** 2016-03-04

**Authors:** Meng Xu, Miao Fang, Yexin Yang, Jaimie T. A. Dick, Hongmei Song, Du Luo, Xidong Mu, Dangen Gu, Jianren Luo, Yinchang Hu

**Affiliations:** ^1^ Pearl River Fisheries Research InstituteChinese Academy of Fishery Sciences/Key Laboratory of Tropical and Subtropical Fishery Resource Application and CultivationMinistry of AgricultureGuangzhou510380China; ^2^ College of Fisheries and Life ScienceShanghai Ocean UniversityShanghai201306China; ^3^ Institute for Global Food SecuritySchool of Biological SciencesQueen's University BelfastMBC, 97 Lisburn RoadBelfastBT9 7BLUK

**Keywords:** Adult sex ratio, generalized multilevel model, nested spatial scales, *Pomacea canaliculata*, variance components

## Abstract

Adult sex ratio (ASR) has critical effects on behavior and life history and has implications for population demography, including the invasiveness of introduced species. ASR exhibits immense variation in nature, yet the scale dependence of this variation is rarely analyzed. In this study, using the generalized multilevel models, we investigated the variation in ASR across multiple nested spatial scales and analyzed the underlying causes for an invasive species, the golden apple snail *Pomacea canaliculata*. We partitioned the variance in ASR to describe the variations at different scales and then included the explanatory variables at the individual and group levels to analyze the potential causes driving the variation in ASR. We firstly determined there is a significant female‐biased ASR for this species when accounting for the spatial and temporal autocorrelations of sampling. We found that, counter to nearly equal distributed variation at plot, habitat and region levels, ASR showed little variation at the town level. Temperature and precipitation at the region level were significantly positively associated with ASR, whereas the individual weight, the density characteristic, and sampling time were not significant factors influencing ASR. Our study suggests that offspring sex ratio of this species may shape the general pattern of ASR in the population level while the environmental variables at the region level translate the unbiased offspring sex ratio to the female‐biased ASR. Future research should consider the implications of climate warming on the female‐biased ASR of this invasive species and thus on invasion pattern.

## Introduction

Adult sex ratio (ASR), defined as the ratio of adult males to adult females in a population, is a central concept in population biology (Bessa‐Gomes et al. 2004; Le Galliard et al. 2005; Ewen et al. 2011; Szekely et al. 2014b). ASR has critical effects on behavior, mate competition and life history and has implications for population demography and biodiversity conservation (Le Galliard et al. 2005; Kokko and Jennions 2008; Liker et al. 2013; Szekely et al. 2014a; Pipoly et al. 2015). For example, with male‐biased ASR, the courtship behavior and male–male competition may intensify (Leftwich et al. 2012); a male‐biased ASR may also increase the rates of aggression and harassment of males to females, which in turn resulting in higher mortality of females (Le Galliard et al. 2005). ASR varies widely among species, ranging from highly male‐biased populations to those composed only of adult females (Donald 2007; Szekely et al. 2014b), and variation across spatial–temporal scales for different populations of a given species (McKellar et al. 2009; Reichard et al. 2014). For instance, Trinidad guppy *Poecilia reticulata* populations in some streams are heavily male‐biased, while others exhibit unbiased or female‐biased ASR (Pettersson et al. 2004); jumping spider *Phidippus clarus* populations shift from male‐biased ASR to a female bias during the breeding season due to the later emergence of females (Hoefler 2007). Understanding the pattern and causes of ASR variance is an important goal in evolutionary biology and population demography given its major role in behavior, life histories, and ultimately population fitness.

Recently, genetic sex determination (Pipoly et al. 2015), sex‐biased survival rate and longevity (Arendt et al. 2014; Szekely et al. 2014a), and environmental conditions (McKellar et al. 2009; Reichard et al. 2014) have been shown to affect the ASR, greatly advancing our understanding of the causes of ASR variation. However, in general, the causes and pattern of ASR variation is still understudied, particularly for variation across spatial scales. The question as to how stable ASRs are in space proposed by Donald (2007) is still poorly understood, which essentially requires analyses of the patterns of ASR across multiple spatial scales and determination of likely causation. Illustrating the scale dependence of ASR variation is important because this could not only provide insight into the mechanisms associated with specific scales, but also shed light on the population dynamics across multiple scales. In the context of biological invasions in particular, sex ratio variation will likely lead to Allee effects, which may strongly influence the establishment and spatial dispersal of invasive population (Engen et al. 2003; Bessa‐Gomes et al. 2004; Tobin et al. 2011; Miller and Inouye 2013). Thus, analyzing the spatial patterns and underlying mechanisms of sex ratio variation can not only enhance our understanding of population biology, but also provide critical information for the management of invasive species.

In this study, we examine the spatial pattern of ASR variation for a highly invasive species, the golden apple snail *Pomacea canaliculata* (Gastropoda, Ampullariidae), across multiple nested spatial scales. *P. canaliculata* is a freshwater snail native to South America and introduced to Zhongshan, Guangdong province, as an aquaculture species in 1981 (Joshi and Sebastian 2006; Yang et al. 2013). This species has invaded a large proportion of South‐East Asian and caused huge economic and ecological damage (Halwart 1994; Cowie 2002; Carlsson et al. 2004; Fang et al. 2010). It has been listed as one of the “Top 100” invasive species by the International Union for Conservation of Nature (Lowe et al. 2000). In the Philippines and Japan, the sex ratio observed in paddy fields is female‐biased with an average ratio of 1:2 (males to females) (Halwart 1994; Tanaka et al. 1999), while the sex ratio of reared populations is 0.5 overall and is found to be determined by a small number of nuclear genes inherited from both parents (Yusa and Suzuki 2003; Yusa 2006, 2007).

Using a multilevel (hierarchical) model framework, we first tested the overall ASR of this invasive species when accounting for sampling independence, then examined the variation in ASR across four spatial scales using variance decomposition and the influence of sampling time. Finally, by adding group‐level predictor variables to the multilevel model, we associated the processes at different scales (e.g., traits of individuals, density in plots, and regional climate characteristics) with this variation to examine potential underlying mechanisms of ASR variation. Specifically, we address following questions: (1) Is there an ASR bias in this invasive species in wild populations? (2) How does ASR vary along nested spatial scales and sampling time? and (3) What biotic and abiotic factors are associated with this ASR variation?

## Materials and Methods

### Data collection

**Figure 1:**
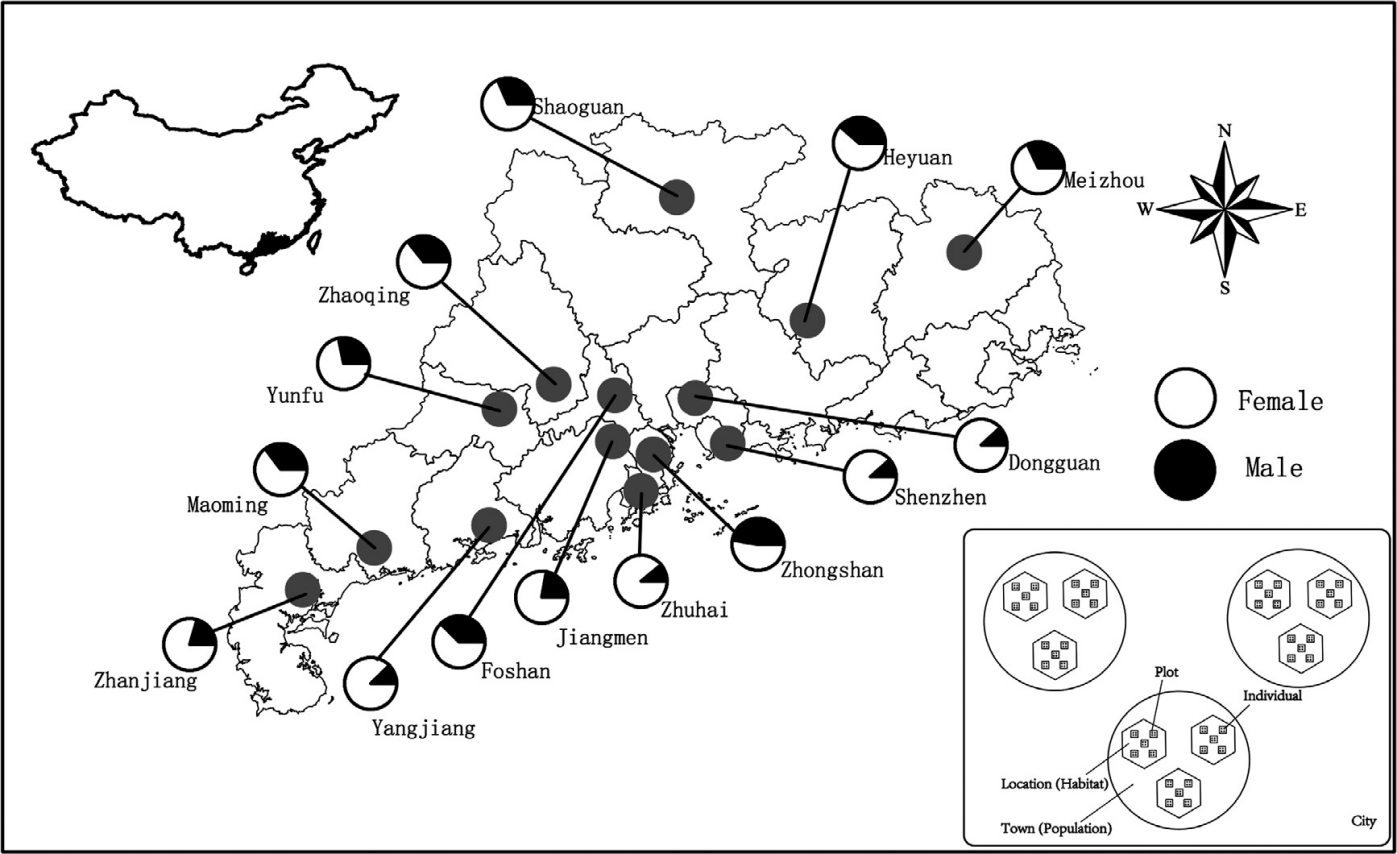
Adult sex ratios of *Pomacea canaliculata* in 14 cities of Guangdong province, China. The gray points on the map do not denote the specific survey location, but the city where we collected samples. In each city, 2–3 towns were chosen randomly (total 35 towns), and in each town, 1–3 habitat sites were chosen randomly (total 60 habitat sites). In each habitat site, 3–5 plots (total 204 plots) were set up. All individuals of this species (body height > 1 cm) in the plot were collected.

This study was conducted in Guangdong province, China, between N20°13′−25°31′ and E109°39′−117°19′, with an annual temperature mean of 22.3°C. In March to June of 2014, surveys and sampling were conducted in 14 cities in Guangdong province, China (Fig. 1: Dongguan, Foshan, Heyuan, Jiangmen, Maoming, Meizhou, Shaoguan, Shenzhen, Yunfu, Yangjian, Zhuhai, Zhanjiang, Zhaoqing, and Zhongshan). These cities represent different regional characteristics that we use to describe the effects of potential climatic conditions such as annual average temperature and precipitation in the multilevel models. In each city, 2–3 towns were chosen randomly to carry out snail surveys (total = 35 towns). These towns are at least 5 km apart from each other, and thus, snails collected can be regarded as independent populations. In each town, 1–3 habitat sites (including wetland, paddy field, ditch et al.) were chosen for sampling plots (total = 60 habitat sites). These sites were used to describe the effects of local habitat characteristics. In each habitat site, 3–5 plots (1 × 1 m) were randomly chosen and the snails (height > 1 cm) in each plot were collected and the density was determined (total = 204 plots). In total, 3424 individual snails were collected and the body weight of each snail (wet weight, with shell) was measured in the laboratory. As offsprings of *P. canaliculata* become mature at about 60 days (Liu et al. 2012) and the size of sexual maturity was about 2.5 cm (Estebenet 1992), we selected those snails with body height larger than 3.0 cm to assess ASR. Under this criterion, 711 individuals in 146 plots, 53 habitats, 34 towns, and 14 cities were chosen for analyses. This nested sampling allows us to partition the variance at each scale to estimate the variation in ASR across multiple spatial scales.

*Pomacea canaliculata* has separate sexes and its sexual dimorphism is well known (Hayes et al. 2015). Females have more globose shells and broader body whorls than males (Martín 2012), are on averagely larger than males (Estebenet and Cazzaniga 1998; Tanaka et al. 1999; Martín and Estebenet 2002), and are heavier than males with the same total length (Estebenet 1998). While the operculum of hatchlings and juveniles of both sexes is concave, the shape of male operculum becomes convex toward the posterior margin when shell length reaches 15–20 mm, whereas those of females remain concave throughout life (Gamarra‐Luques et al. 2013). The sex of each adult snail collected (height > 3 cm) was identified firstly based on curve of the operculum (Halwart 1994), then determined by the presence or absence of testes near the apex (Yusa and Suzuki 2003). The data of annual average temperature and precipitation for each city were gained from the website: http://www.grmc.gov.cn.

### Statistical analysis

Generalized multilevel models (or generalized linear mixed models) can conveniently partition the variance component of multiple scales and account for sampling errors (Gelman and Hill 2007; Qian 2010); thus, in this paper, we use this statistical model to estimate the whole ASR and analyze the variance at different spatio‐temporal scales. Assuming a binomial error distribution and using a logit link function, we firstly fitted a no‐fixed effect multilevel model with the estimated intercept being the log odds (log transformation of the ratio of female to male) of the sex (Qian 2010). The nested scales and sampling time were used as crossed random effects to characterize the sample autocorrelation within the same place and time. We tested whether log odds were significantly different from 0, that is whether the ratio of females to males was significantly different from 1 (Hardy 2002). Then, we reported the ASR as the proportion of females (ASR) through back‐transformation from the intercept. The notation of the model was as follows:

Y∼binomial(P)

$$\text{log}\;\text{it}\;\left(P\right)=\text{log}\left(\frac{P}{1-P}\right)=\beta 0+\text{Random}\;{\text{Effects}}_{\mathrm{city}/\mathrm{town}/\mathrm{habitat}/\mathrm{plot}}+\text{Random}\;{\text{Effects}}_{\mathrm{time}},$$

where *Y* is the binary response (female or male), *P* is the female probability and *P*(1 − *P*) is the ratio of females to males. *β*0 denotes the fixed intercept. Random Effects_city/town/habitat/plot_ and Random Effects_time_ denote random effects for nested spatial scales and sampling time.

By specifically analyzing the Random Effects_city/town/habitat/plot_, we could calculate the variance explained at different spatial scales (variance component analysis), that is, the variation in the ratio of females to males across multiple spatial scales when accounting for the time autocorrelation of samples. In this framework of the generalized multilevel model, the variance was the variation around the mean log ratio of females to males. For any given level, each group mean was first calculated, then the variance of these group means around the group mean of the higher level to which they belong was calculated, then the variance component of each specific level can be gained (Table 1) (Messier et al. 2010; Xu et al. 2015). By analyzing the Random Effects_time_, we also analyzed the variance explained by the sampling time as well.

**Table 1:** Nested ANOVA table for a Type I sum of squares analysis in a hierarchical design. A sex ratio *Y*_*phtc*_ is measured on *p *=* *1…*P* plots per habitat, *h *=* *1…*H* habitats per town, *t *=* *1…*T* towns per city and *c* = 1…*C* cities. $\bar{Y_{htc}}$ is the mean value of habitat *h* of town *t* of city *c*, $\bar{Y_{tc}}$ is the mean value of town *t* of city *c*,* Y*_*c*_ is the mean of city *c*, and $\overset{¯}{Y}$ is the grand mean. The variance components of plot‐, habitat‐, town‐ and city‐level are $\sigma_{P}^{2}$, $\sigma_{H}^{2}$, $\sigma_{T}^{2}$, and $\sigma_{C}^{2}$

Source	Sum squares (SS)	Degrees of freedom (df)	Mean squares (MS)
Among plots within habitat (includes measurement error)	${\text{SS}}_{P}=\sum_{c=1}^{C}\sum_{t=1}^{T}\sum_{h=1}^{H}\sum_{p=1}^{P}{\left(Y_{phtc}-\bar{Y_{htc}}\right)}^{2}$	df_*P*_ = C*T*H*(*P*−1)	${\text{MS}}_{P}=\frac{{\text{SS}}_{P}}{df_{P}}=\sigma_{P}^{2}$
Among habitats within town	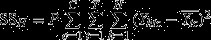	df_*H*_ = C*T*(*H*−1)	${\text{MS}}_{H}=\frac{SS_{H}}{df_{H}}=\sigma_{P}^{2}+P\sigma_{H}^{2}$
Among towns within city	${\text{SS}}_{T}=PH\sum_{c=1}^{C}\sum_{t=1}^{T}{\left(Y_{tc}-\bar{Y_{c}}\right)}^{2}$	df_*T*_ = C*(*T*−1)	${\text{MS}}_{T}=\frac{{\text{SS}}_{T}}{df_{T}}=\sigma_{P}^{2}+P\sigma_{H}^{2}+PH\sigma_{T}^{2}$
Among cities with total	${\text{SS}}_{C}=PHT\sum_{c=1}^{C}{\left(Y_{c}-\bar{Y}\right)}^{2}$	df_*C*_ = *C*–1	${\text{MS}}_{C}=\frac{{\text{SS}}_{C}}{df_{C}}=\sigma_{P}^{2}+P\sigma_{H}^{2}+PH\sigma_{T}^{2}+PHT\sigma_{c}^{2}$
Total	${\text{SS}}_{P}=\sum_{c=1}^{C}\sum_{t=1}^{T}\sum_{h=1}^{H}\sum_{p=1}^{P}{\left(Y_{phtc}-\bar{Y}\right)}^{2}$	df_Total_ = C*T*H*P−1	${\text{MS}}_{\mathrm{Total}}=\frac{{\text{SS}}_{\mathrm{Total}}}{df_{\mathrm{Total}}}=\sigma_{\mathrm{Total}}^{2}=\sigma_{P}^{2}+\sigma_{H}^{2}+\sigma_{T}^{2}+\sigma_{C}^{2}$

To study the potential causes associated with variation in sex ratios at specific spatial scales, we extended the model above by including the individual weight and group‐level predictive variables (snail density in plot level, and temperature and precipitation in city level) into the model. We firstly included all individual‐ and group‐level predictors into the model and tested the significance of fixed effects. The notation of the model including all predictors was as follows:

Y∼binomial(P)

$$\begin{array}{cc} \text{log}\;\text{it}\;(P) & =\text{log}\left(\frac{P}{1-P}\right)\\ & =\beta 0+\beta 1_{\mathrm{trait}}+\beta 2_{\mathrm{density}}+\beta 3_{\mathrm{climate}}\\ & \;+\text{Random}\;{\text{Effects}}_{\mathrm{city}/\mathrm{town}/\mathrm{habitat}/\mathrm{plot}}\\ & \;+\text{Random}\;{\text{Effects}}_{\mathrm{time}}, \end{array}$$

where *Y* is the binary response (female or male) and *P* is the probability of females. *β*0 denotes the fixed intercept and *β*1_trait_, *β*2_density_, and *β*3_climate_ denotes the fixed effect of individual trait, plot density, and city climate characteristics. Random Effects_city/town/habitat/plot_ and Random Effects _time_ denote random effects for nested spatial scales and sampling time. In this model, we focus on testing the fixed effect and consider the unexplained variation in intercepts and slopes merely as error terms.

Then, we, respectively, included each group‐level predictor into an intercept‐varying model and we focus on the variation in ASR at group level and the relationship between group‐level predictors and ASR. The notation of the model was as follows:

Y∼binomial(P)

$$\text{log}\;\text{it}\;\left(P\right)=\text{log}\left(\frac{P}{1-P}\right)=\beta 0+\beta 1_{\mathrm{group}}+\text{Random}\;{\text{Effects}}_{\mathrm{city}/\mathrm{town}/\mathrm{habitat}/\mathrm{plot}}+\text{Random}\;{\text{Effects}}_{\mathrm{time}},$$

where *β*1_group_ is group‐level predictive variables and used to predict the intercept in this multilevel model (Gelman and Hill 2007).

All statistical analyses were conducted using R 3.1.2 (R Core Team 2015). Generalized multilevel models were fitted by the *glmer* function of the “lme4” package (Bolker et al. 2009; Bates 2010). Wald *Z* tests were used to test the fixed effects. The “arm” package was used to extract standard error of model coefficients from objects returned by *glmer* function. The bootstrap method was used to calculate the confidence interval of variance components.

## Results

**Figure 2:**
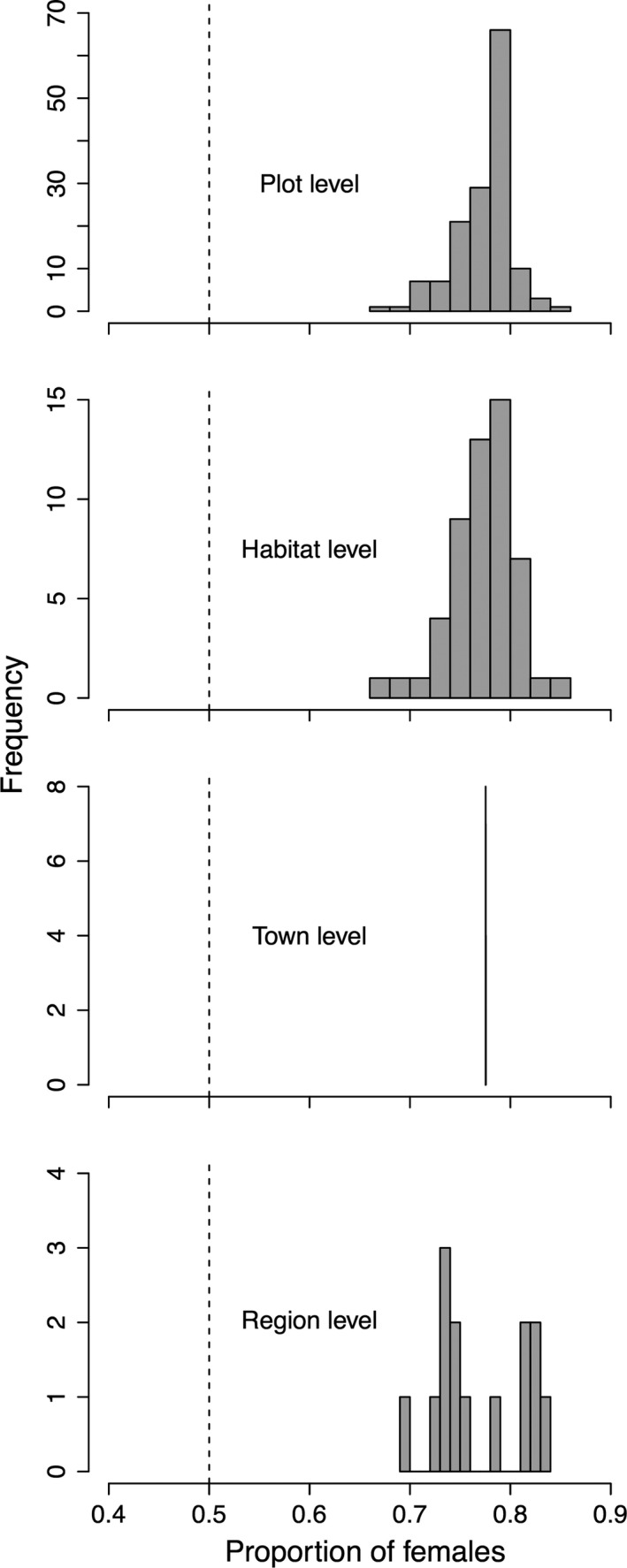
Distributions of adult sex ratio estimated from the generalized multilevel model at four spatial scales.

**Figure 3:**
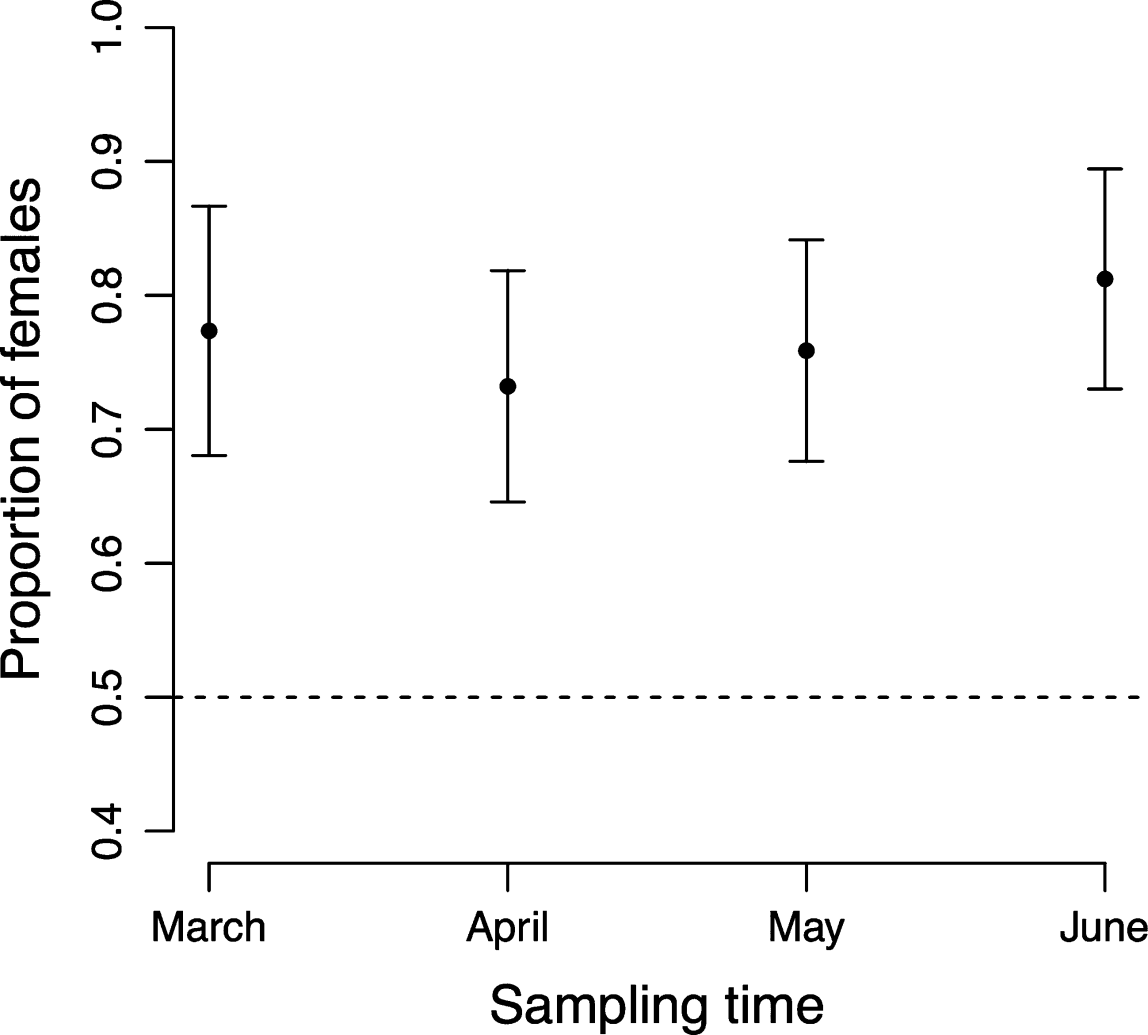
Adult sex ratio estimated from the generalized multilevel model at different sampling months. Dots represent parameter values estimated. Solid lines represent 95% confidence intervals.

**Figure 4:**
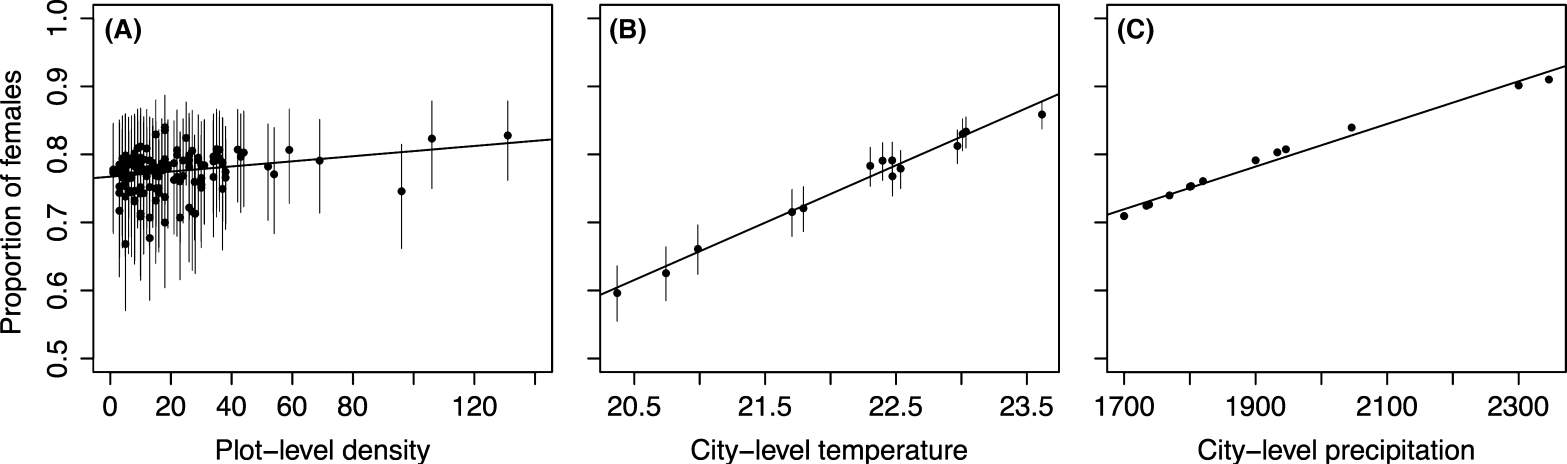
The relationship between (A) density in plot level and adult sex ratio, (B) temperature in city level and adult sex ratio, and (C) precipitation in city level and adult sex ratio, respectively. Dots represent the parameter values (±1SE) estimated from the generalized multilevel model including group‐level predictors.

The ASR of the golden apple snail *P. canaliculata* was significantly female‐biased, with the proportion of females being 0.78 (Table 2, Figs. 1, 2). The partitioning of variance reveals fairly balanced distributions of variance across three of the four spatial scales, with 31.32%, 27.93%, and 28.06% variation of ASR occurring in the plot, habitat, and city scales, respectively (Table 2, Figs. 2, S1). However, the town level stands out as containing none of the total variance. Sampling time explained 12.69% of the variation of ASR (Table 2, Fig. 3). Individual weight and plot‐level density did not significantly associate with ASR (Table 3, Fig. 4). The city (region‐level) temperature and precipitation significantly associated with the ASR, with increasing temperature and precipitation associating positively with the proportion of adult female snails (Table 3, Fig. 4). The extreme low temperature in region revel was also significantly associated with the ASR (Fig. S2).

**Table 2:** Results of a generalized multilevel model with nested spatial scales (plot, habitat, town, and city) and sampling time (months) as random effects, but without fixed effect. This model aimed to (1) test the total intercept (log ratio of females to males) when accounting for the spatial and temporal autocorrelation of samples and (2) analyze the variance component explained at different spatial and temporal scales. 95% confidence intervals were obtained through bootstrap method (999 runs with 711 randomly sampled data points with replacement). Significant results are shown in boldface type

Fixed effect	Estimate (SE)	*Z*‐value	*P* (>|*z*|)
Intercept	**1.239** (**0.252**)	4.920	<**0.001**
Random effects	Variance	95% CI	
Plot: (habitat: (town: city))	**0.211**	[0.105, 0.989]	
Habitat: (town: city)	**0.184**	[0.110, 1.027]	
Town: city	0	[−0.543, 0.248]	
City	**0.185**	[0.087, 1.042]	
Time	**0.076**	[0.017, 0.810]	

**Table 3:** Results of generalized multilevel model including weight as individual‐level predictive variables, and density, temperature, and precipitation as group‐level predictive variables. In this model, nested spatial scales (plot, habitat, town, and city) and sampling time (months) were included as random effects. Significant results are shown in boldface type

Fixed effects	Estimates (SE)	*Z*‐value	*P* (>|*z*|)
Intercept	−**11.730** (**3.996**)	−2.935	**0.003**
Weight	−0.009 (0.02)	−0.443	0.665
Density	−0.002 (0.006)	−0.330	0.741
Temperature	**0.401** (**0.159**)	2.526	**0.012**
Precipitation	**0.002** (**0.001**)	2.836	**0.005**

## Discussion

Using a field survey dataset for the invasive golden apple snail *P. canaliculata* in China, we examined the ASR, quantified its variation across multiple spatial and temporal scales, and analyzed the potential associations between ASR and abiotic/biotic factors. Taken together, we found that the ASR of this species was significantly female‐biased and that the female‐biased ASR was equally distributed across three of four nested spatial scales while no variance was found at the town level. Individual traits and density at the plot level were not correlated with the ASR, while the temperature and precipitation at the region level was significantly positively associated with the variation of ASR.

### Female‐bias and spatial–temporal variation in ASR

We found that the ASR of this invasive species was strongly female‐biased. For past 20 years, the female‐biased ASR of this highly invasive species *P. canaliculata* has been found consistently in China (Zhang et al. 1993; Yang et al. 1994; Yin et al. 1999; Liu et al. 2012; Qin et al. 2012; Guo et al. 2014). We verified this here and found that the proportion of the adult females can reach 78% when statistically controlling for the sampling spatial–temporal autocorrelation. In the Philippines and Japan, the sex ratio of *P. canaliculata* in the field is also female‐biased with an average ratio of 1:2 (males to females) (Halwart 1994; Tanaka et al. 1999), while in Thailand the bias of sex ratio was found changed with season during 1 year (Banpavichit et al. 1994). However, the offspring sex ratio of this species has been found to be unbiased and to be determined by a small number of nuclear genes inherited from both parents (Yusa and Suzuki 2003; Yusa 2006, 2007). It seems that some biotic or abiotic factors differentially influence the females and males in the growth stage, shifting the balance of offspring sex ratio of *P. canaliculata* to a female‐biased ASR. However, the sampling criterion (height > 3 cm) and the resulting relative low sample size (711 adults) may exert some influences on our result. Although the relatively large adults selected can increase the accuracy of distinguishing sex, the relative small sample size may also increase the random variance, particularly at the plot and habitat levels. Meanwhile, given that the mature males may have smaller body size than female adults, the sampling criterion may underestimate the proportion of males due to artificially excluding small male adults.

Adult sex ratio may vary among different locations if females and males response differently to biotic/abiotic variables among these locations. For example, in the Trinidad guppy *Poecilia reticulata,* populations in some streams had male‐biased ASR, whereas others exhibit unbiased or female‐biased ASR (Pettersson et al. 2004). Sex‐differential sensitivity to high temperature and predators may drive this phenomenon (Pettersson et al. 2004; McKellar et al. 2009). Woolfenden et al. (2001) found that the ASR of the Brown‐headed Cowbird *Molothrus ater* was balanced in eastern USA, whereas it was male‐skewed in western USA. However, except for these studies, spatial variation of ASR is understudied, particularly with regard to variation across multiple scales. Our variance component analysis indicated that ASR variance of *P. canaliculata* is fairly evenly distributed across spatial plot, habitat, and city levels, suggesting that processes at these scales probably are equally important in determining ASR. In general, several processes (including different offspring sex ratio, sampling bias, sex difference in survival, migration, age at maturity) may result in a biased ASR (Arendt et al. 2014). In the 1 × 1 m plot level, the variance captured the variation between plots and census error. The density of the plot probably differentially affected the survival, migration, and lifespans of the females and the males. As the brood sex ratio was highly variable, the plot may capture the variation of brood level driven by genetic factors (Yusa and Suzuki 2003; Yusa 2004, 2007). At the habitat scale, variation of ASR explained as much as at the plot scale, indicating that the local abiotic/biotic factors such as physical and chemical characteristics of water, the type, and palatability of food and occurrence of natural enemies may influence the ASR. Some studies have also shown that female and male *P. canaliculata* differ in foraging, heterosexual choice, and alarm response to predator (Xu et al. 2011, 2014). The variation of ASR in the city scale illustrated the importance of climatic characteristic such as temperature, precipitation, light intensity, and photoperiod in influencing the ASR. Considering the relatively uniform distribution of the variance among three of the four spatial scales, the main ecological and evolutionary processes in these scales ought to be taken into account in the future to more thoroughly analyze the causes of ASR variation.

In contrast to the other three levels, the town levels surprisingly accounts for none of the total variation in ASR, although they were all female‐biased. The towns in each city were at least several kilometers apart from each other, and thus, they could be viewed as independent populations. This indicated that ASR did not change at this specific population level (within city but above habitat and plot) and some process at this level was not related to the ASR. Several reasons may result in this situation. If some environmental factors such as temperature or precipitation at larger scale were the main contributors to the variation of ASR, the small variation of them in the different towns of one city should lead to equal distribution of ASR at this level. Our result with respect to ASR was consistent with the previous experimental investigation in the laboratory that offspring sex ratio was unchanged among populations, but highly variable among broods within populations (Yusa and Suzuki 2003). Unlike our finding, however, these authors found offspring sex ratio in the population‐level was not significantly biased from 0.5.

Adult sex ratio may also vary in time; for instance, more common male migratory birds experienced stronger competition for mates, leading to earlier arrival and thus male‐biased sex ratios in the early stage (Kokko et al. 2006). In a recent study, the ASR of the guppy *P. reticulate* varied dramatically throughout the year due to sex differences in adult survival rates and longevity (Arendt et al. 2014). In our study, we did not detect significant variation of ASR in *P. canaliculata* in different census months. Similarly, a recent study also found that ASR for three of four African annual fish did not change with the census season (Reichard et al. 2014). However, the short census time may also hinder detection of mortality of adult snails and the recruitment of juveniles given that we tested the time trend of just 4 months. To analyze the variation of ASR over time for this invasive species, census across different seasons and years is required in the future.

### Causes and implications of skewed ASR

In general, variation in ASR may be due to four mechanisms. First, skewed ASR may emerge as a consequence of skewed offspring sex ratio. Before maturation, sex ratio may be already biased at conception (primary sex ratio), or at birth and hatching (secondary sex ratio) (Clutton‐Brock 1991; West 2009). For *P. canaliculata*, an elegant study has found that the average offspring sex ratio was balanced (Yusa and Suzuki 2003). Thus, rather than offspring sex ratio, other physiological, ecological, and evolutionary process may shape the current ASR of this species in the field. Second, sex differences in adult mortality rate may lead to skewed ASRs. When one sex suffers more predation, parasites, or pathogens (Berger and Gompper 1999; Hirst et al. 2010), or stronger intraspecies competition for limited resources, or when it was more susceptible to some environmental conditions, skewed ASRs may occur. *Pomacea canaliculata* can be consumed by a wide array of insect, crustaceans, fish, reptiles, leeches, birds, and mammals in the field at the juvenile stage (Hayes et al. 2015). In, particular, the carp, tilapia, ducks, and turtles have been evaluated as effective biocontrol agents, although their use probably impacted some native aquatic species as well (Yoshie and Yusa 2008; Wong et al. 2009). Additionally, a large number of endosymbiotic animals, protists, and bacteria have also been found to establish in the *P. canaliculata*, which probably differentially influence the females and males (Hayes et al. 2015). In this study, however, we focused on the macroscale analysis of ASR and the potential effects of predators and parasites were not examined. We examined the density effect, probably reflecting resource competition on the ASR, and found no evidence that this factor could contribute to the current pattern of skewed ASR. Third, sex‐differential maturation times may result in an excess of the sex that matures earlier. A rearing experiment in China indeed found that average maturation time of females was shorter than that of males (Liu et al. 2012). Meanwhile, the age at maturation of *P. canaliculata* may also be affected by food availability, population density, and abiotic environment (Tanaka et al. 1999; Estoy et al. 2002; Hayes et al. 2015). Fourth, different movement pattern may also cause skewed ASR. As one sex moves out from a population, then it must move into another population; however, this possible migration difference will only affect the local ASR rather than the population ASR. For our study, thus, migration could not explain the skewed ASR, as ASR was always female‐biased irrespective of any spatial level.

We did find that annual average temperature, extreme low temperature, and average precipitation in the city level had significant associations with ASR. The ASR increased significantly with the increasing temperature and precipitation, indicating the abiotic factors in the field can adjust the ASR of this species. Combing our current finding with previous result (Yusa and Suzuki 2003), we suggest that offspring sex ratio in this species may shape the general pattern of ASR in the population level when the regional environmental characteristics translate the unbiased offspring sex ratio to the female‐biased ASR. More research on other taxa should be conducted to test whether this phenomenon is pervasive and, if possible, more ecological scales should be included in the analysis to separate the variation of ASR and to infer the potential mechanisms. In the context of biological invasions, more females could mean more rapid population growth and hence potential spread, but that lack of males could also lead to infertile females (i.e., Allee effects). More studies regarding the implications of skewed ASR on the invasive dynamics are required. In particular, as we found that the increasing temperature could intensify the female bias, the potential implications of climate warming on ASR and hence invasion pattern should be paid more attention.

## Conclusion and Future Direction

In this paper, we analyzed the spatial variation of ASR across multiple scales and found that at some specific spatial scale ASR may show little variation. We determined that ASR of this species was female‐biased in field and that environmental variables at the regional level were important predictors for the ASR of this species. Our study may provide a general model to study the trait variations across multiple scales. More studies are required to explore the population development responding to this skewed ASR. For invasive species, it may also have important implications on the causes and implication of species invasions to analyze the variation of ASR between invasive and native ranges and along invasion timelines.

## Conflict of Interest

None declared.

## Supporting information


**Figure S1.** Adult sex ratio estimated from the generalized multilevel model at four spatial scales.
**Figure S2.** The relationship between extreme low temperature and adult sex ratio at the city level.
