# Supplementary material for: Spatial variation in adult sex ratio across multiple scales in the invasive golden apple snail, Pomacea canaliculata
Source: Ecol Evol. 2016 Mar 4;6(8):2308–17. doi: 10.1002/ece3.2043 (PMC4782258; doi:10.1002/ece3.2043)
Supplement: Supplementary file 1 — Figure S1. Adult sex ratio estimated from the generalized multilevel model at four spatial scales. Figure S2. The relationship between extreme low temperature and adult sex ratio at the city level. [file ECE3-6-2308-s001.doc]

**Supplementary materials:**

**Figure S1.** Adult sex ratio estimated from the generalized multilevel model at four spatial scales. Dots represent parameter values estimated. Solid lines represent 95% confidence intervals.

**Figure S2.** The relationship between extreme low temperature and adult sex ratio at the city level. Dots represent the parameter values (±1SE) estimated from the generalized multilevel model including group level predictors.
